# Culture of East Indian sandalwood tree somatic embryos in air-lift bioreactors for production of santalols, phenolics and arabinogalactan proteins

**DOI:** 10.1093/aobpla/plt025

**Published:** 2013-05-06

**Authors:** Biswapriya B. Misra, Satyahari Dey

**Affiliations:** Plant Biotechnology Laboratory, Department of Biotechnology, Indian Institute of Technology Kharagpur, Midnapore (West), Kharagpur 721302, West Bengal, India

**Keywords:** Air-lift bioreactor, arabinogalactan protein, phenolics, santalol, *Santalum album*

## Abstract

The East Indian sandalwood tree, *Santalum album*, yields one of the costliest heartwoods and precious essential oil. Unsurprisingly, this endangered forest species is severely affected due to unmet global demands, illegal trade and harvesting, overharvesting and an epidemic mycoplasmal spike disease. *In vitro* micropropagation endeavours have resulted in defined *in vitro* stages such as somatic embryos that are amenable to mass production in bioreactors. We report on somatic embryo production in a 10-L air-lift-type bioreactor, and compare the growth and biochemical parameters with those of a 2-L air-lift-type bioreactor. For the 10-L bioreactor with biomass (475.7 ± 18 g fresh weight; *P* < 0.01), concomitantly santalols (5.2 ± 0.15 mg L^−1^; *P* < 0.05), phenolics (31 ± 1.6 mg L^−1^) and arabinogalactan proteins (AGPs) (39 ± 3.1 mg L^−1^; *P* < 0.05) are produced in 28 days. In addition, we identified and quantified several santalols and phenolics by means of high-performance thin-layer chromatography and reverse-phase high-pressure liquid chromatography analyses, respectively. Results indicate that 10-L-capacity air-lift bioreactors are capable of supporting somatic embryo cultures, while the extracellular medium provides opportunities for production of industrial raw materials such as santalols, phenolics and AGPs. This will prove useful for further optimization and scale-up studies of plant-produced metabolites.

## Abstract (precis)

The Indian Sandalwood tree is globally acclaimed for the precious essential oil and heartwood. Over-exploitation, diseases, and habitat loss have posed significant challenges to find an alternative bioresource for biomass production. Here, we report the successful growth of *in vitro* grown somatic embryos in 10 L air-lift bioreactors. Additionally, we characterized arabinogalactan proteins and small molecule constituents such as phenolics and terpenoids that are secreted by the suspended somatic embryos into the culture media. In parallel to the biochemical characterisation, we followed the entire developmental progression of proembryogenic masses into matured cotyledonary embryos during a single run of the bioreactor.

## Introduction

The East Indian sandalwood tree, *Santalum album*, is a woody and tropical forest tree that belongs to the family Santalaceae. The species is globally acclaimed for its very costly heartwood and essential oil obtained from matured individuals. The steam-distilled commercially available essential oil is rich in sesquiterpenoid constituents known as ‘santalols’, i.e. α-santalol and β-santalol together contributing to >90 % (Howes *et al.* 2004). Unmet anthropogenic needs for use in perfumery (fragrance) and as food additives (flavour) have led to the decline of natural populations due to illegal trade and harvesting, and overexploitation. The estimated global annual requirement is ∼10 000 tons of wood (equivalent to 200 tons of oil), involving a trade of approximately $125 million, of which only 10 % is met from natural resources. Its natural enemy in the form of the mycoplasmal ‘spike disease’ has led the species into the IUCN (International Union for Conservation of Nature and Natural Resources) Red List of Threatened Species (IUCN 2012). Sandalwood finds extensive applications in traditional medicinal systems such as Ayurveda, and is gaining increasing importance in modern pharmacological investigations as a source of anticancer (Bommareddy *et al.* 2012), anti-*Helicobacter pylori* (Ochi *et al.* 2005) and antiviral (Benencia and Courreges 1999) biomolecules. However, the havoc caused by the epidemic spike disease and the hemi-parasitic and slow-growing nature of the tree necessitated research towards the development of biotechnological means of *in vitro* production as early as 1963, i.e. callusing (Rangaswamy and Rao 1963). Further efforts on somatic embryo (SE) production (Bapat *et al.* 1990) and maturation in air-lift bioreactors (Das *et al.* 1998, 1999) have also been successful.

Plant cell cultures have yielded valuable natural products in the form of pharmaceuticals, flavours and fragrances, and agricultural, cosmetic, bioherbicidal and fine chemicals, with ∼2000 new plant chemicals added annually. The global market for plant-derived drugs was worth an estimated US$18 billion in 2005, with an expected annual growth rate of 6.6 % to US$26 billion by 2011, with the USA accounting for 50 % of the global plant-derived drug market (Saklani and Kutty 2008). These phytomolecules are proven immunomodulating, antiviral, antimicrobial, antiparasite, antitumour, anti-inflammatory, hypoglycaemic, tranquillizer and antifeedant agents. Furthermore, the homogeneous and synchronous nature of cell suspension cultures makes them amenable to large-scale production of phytochemicals. Successful reports include those from Japan on the production of shikonin, ginseng and berberine at commercial scales using bioreactors (Misawa 1991). In 1984, shikonin was the first product that was produced from cell cultures of *Lithospermum erythrorhizon* in 750-L bioreactors by Mitsui Petrochemical Co., Japan (Tabata and Fujita 1985). Advances made towards commercialization of plant cell culture processes for the synthesis of biomolecules have been reviewed recently (Wilson and Roberts 2012). To this end, excellent reviews have been published underscoring the efficient production of secondary metabolites (Kolewe *et al.* 2008; Weathers *et al.* 2010) and recombinant proteins (Huang and McDonald 2009), with an emphasis on the development of suitable bioreactor configurations for plant cell culture-based processes (Georgiev *et al.* 2009). Additionally, somatic embryogenesis offers a potential system for large-scale plant propagation in automated bioreactors (Paek *et al.* 2005). Furthermore, disposable bioreactors have been used for micropropagation of plant tissues, undifferentiated bioactive cells and for expression of secondary metabolites and glycoproteins (Eibl *et al.* 2009). Similarly, successful product/platform pairs leading to synergies during production and in clinical trials have also been reviewed (Paul and Ma 2011). Additionally, polyphenols and isoprenoids have been identified as major classes of antioxidants produced by the plant cells *in vitro* and were reviewed (Matkowski 2008). Similarly, Xu *et al.* (2011) have identified the use of arabinogalactan proteins (AGPs) as ‘tags’ for facilitation of production of recombinant pharmaceutical proteins in plant cell suspension cultures on a large scale. Additionally, somatic embryogenesis offers a potential system for large-scale plant propagation in automated bioreactors (Paek *et al.* 2005).

Thus, to meet the challenges of the commercial viability of *S. album*, a protocol with high and consistent production of SEs is necessary. To this end, a bioreactor-based production system using a liquid medium was developed (Das *et al.* 1998). However, the scale-up study reported earlier (Das *et al.* 1999) did not address the detailed investigation of biochemicals. In this study, we compare the culture of SEs in 2- and 10-L air-lift bioreactors as a biotechnological means of propagation for this endangered species in terms of the growth and biochemical characteristics.

## Methods

### Bioreactors

A 2-L-volume air-lift bioreactor (Bio Stat, B. Braun Biotech. International, Lancaster, PA, USA) and a 10-L culture vessel (Cat. No.: 2227-0020, Nalgene Nunc™ International, Rochester, NY, USA) were used in this investigation. The 10-L culture vessel (Nalgene) was modified to perform as an air-lift-type bioreactor. For comparison, the earlier reported 2-L (Bio Stat, B. Braun) air-lift bioreactor (Pal *et al.* 2003) was studied for growth of sandalwood SEs. For the 10-L vessel we used a modified internal draft tube of 20 mm diameter attached to silicone tubing for air passage through 0.21-µm filters as inlet and outlet. The constant aeration sources were two oil-free air compressors. Agitation was maintained at a constant rate, i.e. 4 L min^−1^ of air flow. Air-lift through an internal draft tube allowed the cultures to agitate at 60–80 rpm. The ambient temperature of the laboratory was maintained at 25 ± 2 °C. The light conditions were set at 3000 lux (16-h light/8-h dark cycles). The data presented in this study are the outcome of four independent runs conducted over several weeks.

### Plant materials

Callus developed from a highly proliferating cell line (IITKGP/91) was maintained in the laboratory as suspension cultures in liquid media, i.e. WPM (Woody Plant Medium) media (Lloyd and McCown 1981) supplemented with 100 mg L^−1^ myo-inositol, 1 mg L^−1^ (4.52 mM) 2,4-dichlorophenoxyacetic acid and 3 % sucrose. Cultures were maintained by subculturing 2-week-old suspensions by transferring each culture into five 250-mL-volume Erlenmeyer flasks containing 50 mL of fresh media. Somatic embryogenesis in suspension was induced in 2-week-old callus by transferring into WPM media supplemented with 100 mg L^−1^ myo-inositol, 0.5 mg L^−1^ (2.85 mM) indole-3-acetic acid, 0.5 mg L^−1^ (3.99 mM) 6-benzylaminopurine and 3 % sucrose and subculturing as described above for 3–6 weeks. Suspension cultures were maintained at 25 ± 2 °C on an orbital shaker (C-24 incubator shaker, New Brunswick Scientific, USA) at 120 rpm under cool-white fluorescent lighting (1500 lux) with a 16-h day/8-h night daily cycle.

### Preparation of inoculum

The bioreactors carrying 1.5 and 7.5 L of WPM media (as described in the ‘Plant materials’ subsection) were sterilized in vertical autoclaves for 35 and 45 min, respectively, at 121 °C and 15-psi pressures. The rest of the media were sterilized separately and allowed to cool down, and fresh inoculum was mixed with sterile media in a laminar air flow cabinet. Two-week-old suspension cultures yielding the proembryogenic mass (PEM) of cells were used as the inoculum for both the air-lift-type bioreactors at a dose of 2 % (v/v) fresh weight (FW).

### Growth rate and biomass estimation

The specific growth rate (μ) and doubling time (*t*_d_) of SE suspension culture were calculated with the following formulae: *μ* = (ln DW_2_ − ln DW_1_)/(*T*_2_ − *T*_1_) and *t*_d_ = ln 2*μ*^−1^, where DW_1_ and DW_2_ are corresponding dry weight masses in days and during exponential growth, and T1 and T2 are the culture initiation and harvest days in numbers.

**Figure 1.**
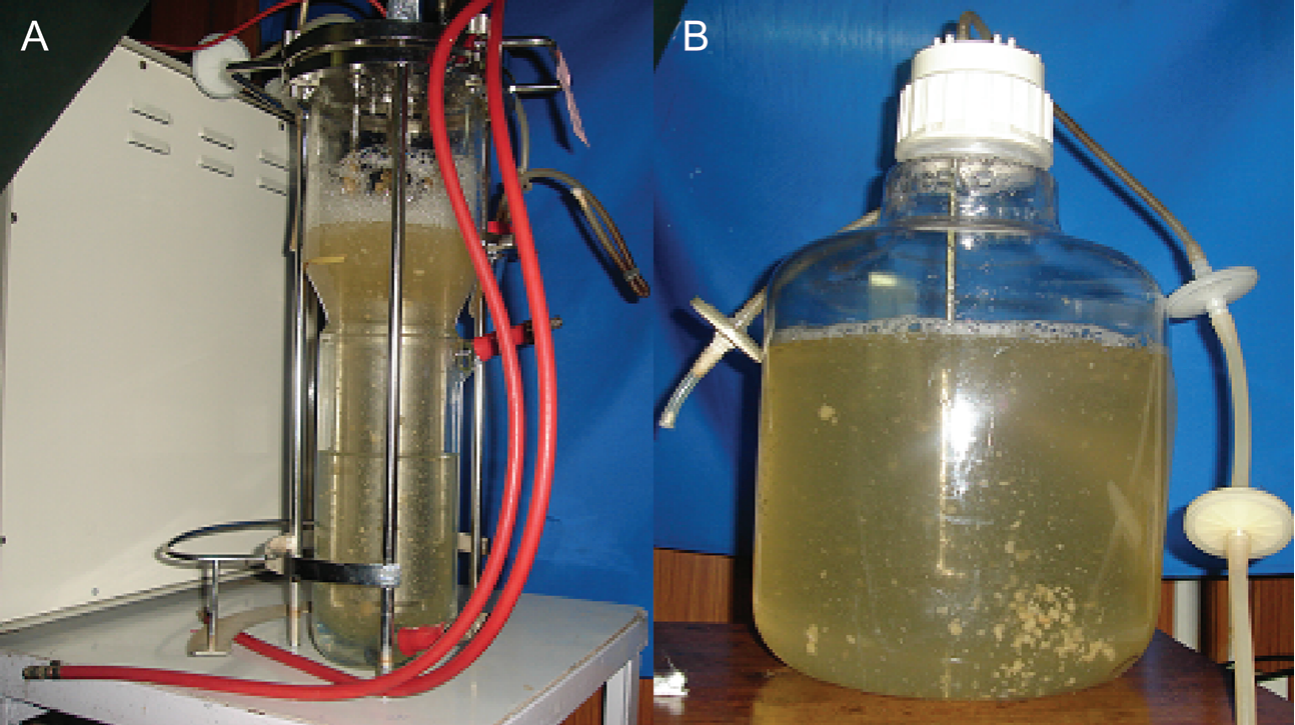
Sandalwood SE cultivation in 2-L (A) and 10-L (B) air-lift bioreactors.

For FW determination, the suspensions were filtered with a Whatman No. 1 filter paper, gently pressed on filter papers to remove excess water and weighed. Cells were dried in an oven at 60 °C to a constant weight for the determination of cell DW. The initial culture volumes were 2 and 10 L, respectively, for the B. Braun and the Nalgene air-lift reactors (Fig. 1A and B) **[see Supporting Information – Videos]**. Growth in terms of biomass was expressed as FW and DW upon harvesting at the end of 28 days, for the 2- and 10-L bioreactors, respectively.

### Determination of SE counts

Although the embryogenic suspension also contained embryos of other stages, the majority were globular stage embryos. Data were recorded on the basis of number of globular embryos per 50 mL of medium produced at the end of 14 days of culture. The number of globular embryos was counted manually for representative smaller aliquots under a Leica Wild (M3Z) (Leica, Wetzlar, Germany) stereomicroscope.

### Extraction of santalols from extracellular medium

The sesquiterpenoids were extracted following protocols described elsewhere (Harborne 1998) and were further analysed by high-performance thin-layer chromatography (HPTLC).

### HPTLC analysis for santalols

Sesquiterpenoids from the suspension culture extracts were analysed by HPTLC as described previously (Misra and Dey 2012). Sesquiterpenoids were identified in comparison with constituents obtained from authentic steam-distilled sandalwood oil samples from Cauvery™ (Government of Karnataka, India).

### Extraction of phenolics from extracellular medium

The extracellular medium (ECM) was collected, filtered with four layers of miracloth, then with laboratory-grade filter paper and finally with Whatman No. 1 filter paper, and then centrifuged at 5000 *g* for 20 min to remove the particulate matter. This was repeatedly extracted three times with diethyl ether using a separatory funnel in a ratio of 1 : 1 of solvent and ECM. The diethyl ether fraction was evaporated to dryness under vacuum at 37 °C and redissolved in 50 % aqueous methanol to yield the phenolics-rich fraction. The ECM thus extracted was saponified with 2 N NaOH at pH 2 for 24 h to release the ester- and ether-linked phenolics, neutralized with 2 N HCl, and then re-extracted as above and pooled together for quantification as well as reverse-phase high-pressure liquid chromatography (RP-HPLC) analysis.

### Folin–Ciocalteau assay for quantification of phenolics

The total phenolic content of the ECM was quantified as described previously (Julkunen-Tiitto *et al.* 2005). Phenolic content was determined using the Folin–Ciocalteau assay and a standard curve prepared with gallic acid.

### RP-HPLC of phenolics

Reverse-phase high-pressure liquid chromatography analyses were performed on a Phenomenex™ (Torrance, CA, USA) C18 column (RP-Hydro, 4 µm, 250 × 4.6 mm) using a Waters HPLC system (Milford, PA, USA) equipped with a dual-absorbance UV detector. Chromatograms were monitored simultaneously at 254 and 310 nm and analysed on a Windows XP™ platform with BREEZE™ software version 3.2 (Waters), and separations were achieved following the method of Sachan *et al.* (2004). The phenolic acids were identified by comparing their retention times with those from authentic standards and co-elution experiments.

### Extraction and quantification of AGPs in ECM

After clarification of the ECM obtained from suspension cultures by high-speed centrifugation, AGPs were precipitated by excess Yariv reagent in 2 % v/v NaCl. The Yariv–AGP complex was then dissociated via reductive cleavage of the diazo linkage with sodium dithionite, followed by dialysis and freeze-drying as described previously (Pal *et al.* 2003). Arabinogalactan protein was determined using β-glucosyl Yariv reagent binding in a single radial gel diffusion assay (Kitazawa *et al.* 2013). Yariv reagent was synthesized in the authors' laboratory as described previously (Pal *et al.* 2003).

### Histology and microscopy of SEs

The SEs collected after final harvest were observed under a Wild (M3Z) (Leica, Wetzlar, Germany) stereomicroscope at ×20 magnification and compared. Wiesner staining of whole SEs for the detection of lignin was performed in 1 % phloroglucinol (prepared in 92 % ethanol) for 2 min (Pomar *et al.* 2002).

### Statistical analyses

Statistical analysis was performed with the SPSS software package (version 17) (SPSS Inc., Chicago, IL, USA). Statistical analyses were performed by Student's *t*-test. *P* values less than 0.05 (*) and 0.01 (**) were considered to be statistically significant. Results were expressed as mean ± SD (error bars) for data obtained from four independent batches of bioreactor run.

## Results

### Yield of biomass from the bioreactors

There were some heart and torpedo stage embryos in most of the batches (<1 % of the number of globular embryos) and these were neglected in the counts for normal and abnormal embryos. The total SE yield in terms of count was 3200 and 5500 L^−1^ of medium for the 2- and 10-L reactors, respectively. Post-cultivation, the media volumes of the 2- and 10-L reactors were reduced to 65 and 75 % of starting culture volumes, respectively. In addition, the pH had decreased to 4.2–4.6 at the time of final harvest. The biomass yields (both in FW and DW) were 2.9- to 3-fold higher for the 10-L bioreactor compared with the 2-L bioreactor. The specific growth rate (*μ*) was 0.0675 and 0.0549 day^−1^, and the doubling time (*t*_d_) was 10.26 and 12.62 days, for the 2- and 10-L air-lift reactors, respectively (Table 1).

**Table 1.** Comparison of growth parameters and biochemical changes for the two air-lift bioreactors. ^a^Values are means of four independent experiments represented as ±SD. ^b^Constant aeration from an oil-free air compressor. *P < 0.05, **P < 0.01.

Reactor-type parameters^a^	B. Braun	Nalgene
Initial culture vol. (L)	2	10
Final culture vol. (L)	1.3 ± 0.5	7.5 ± 0.3*
Inoculum (% w/v, FW)	2	2
Air flow rate^b^ (L min^−1^)	4	4
Air-lift through internal draft tube (rpm)	60	80
Cultivation period (days)	28	28
Temperature (°C)	25 ± 2	25 ± 2
Initial pH	5.1 ± 0.25	5.3 ± 0.2
Final pH	4.2 ± 0.25	4.6 ± 0.3
Biomass yield (g FW)	155.3 ± 6.5	475.7 ± 18**
Biomass yield (g DW)	28.4 ± 3.5	82.5 ± 7.5**
Specific growth rate (*μ*) (day^−1^)	0.0675	0.0549*
Doubling time (*t*_d_) (days)	10.26	12.62*
Santalols (mg L^−1^)	4.3 ± 0.19	5.2 ± 0.15*
AGP yield (mg L^−1^)	33 ± 2.8	39 ± 3.1*
Phenolics yield (mg L^−1^)	29 ± 1.2	31 ± 1.6

### RP-HPLC-based identification of ECM phenolics

**Figure 2.**
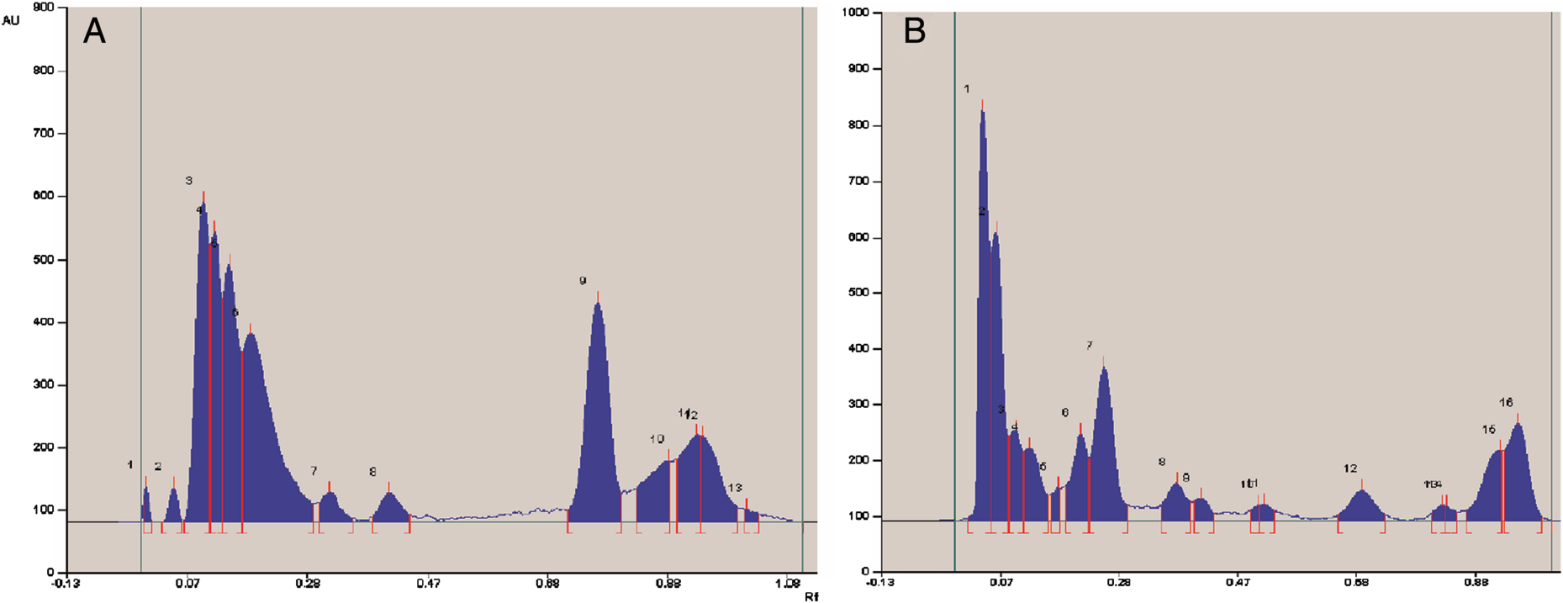
High-performance thin-layer chromatography profiles of sesquiterpenoids obtained from the ECM of 2-L (A) and 10-L (B) air-lift bioreactors.

Various types of phenolics obtained from the ECM of SEs were identified by RP-HPLC (Table 2). Air-lift bioreactor-grown SE culture ECM showed the presence of several phenolics, i.e. 4-hydroxybenzyl alcohol, 4-hydroxybenzaldehyde, and protocatechuic, *p*-coumaric and ferulic acids. Only vanillin was detected exclusively in the ECM of the 10-L bioreactor. Moreover, the RP-HPLC chromatograms indicated that 15–19 phenolic constituents remained unidentified in both the ECM samples (Fig. 2A and B).

**Table 2.** Identification and quantification of phenolic constituents from the air-lift bioreactors as inferred from RP-HPLC analyses. n.d., not detected.

Serial no.	Phenolic constituents identified	Phenolic content (mg L^−1^ ECM)	
		2-L B. Braun reactor	10-L Nalgene vessel
1	4-Hydroxybenzyl alcohol	0.55 ± 0.09	0.75 ± 0.13
2	Protocatechuic acid	0.12 ± 0.02	0.11 ± 0.03
3	4-Hydroxybenzaldehyde	0.87 ± 0.17	0.75 ± 0.12
4	*p*-Coumaric acid	1.13 ± 0.21	1.57 ± 0.35
5	Ferulic acid	0.33 ± 0.08	0.46 ± 0.11
6	Vanillin	n.d.	0.35 ± 0.06
7	Unidentified phenolic constituents	19	15

### HPTLC-based analysis for santalols

**Figure 3.**
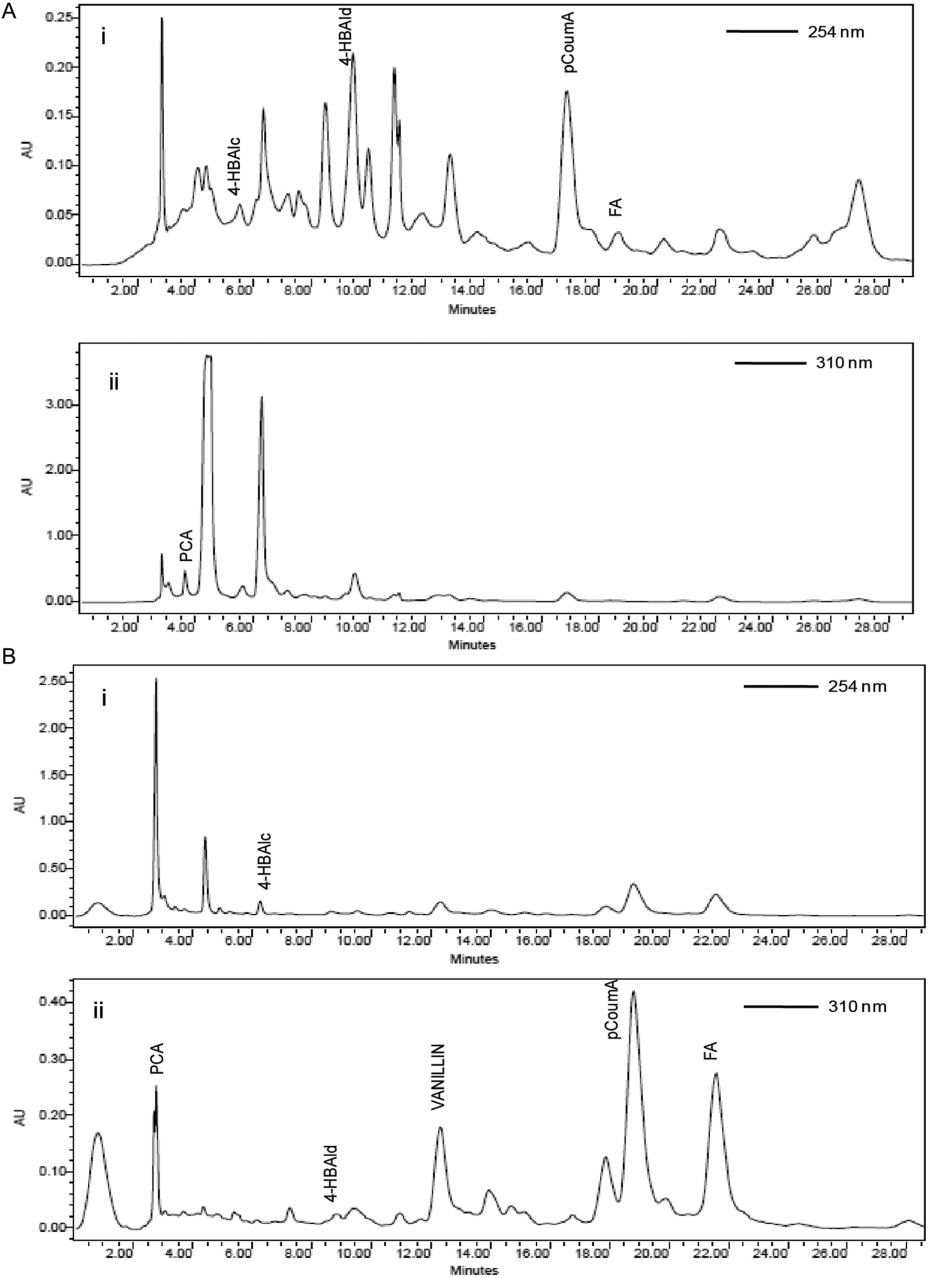
Reverse-phase high-pressure liquid chromatography profile of phenolics obtained from the ECM of 2-L (A) and 10-L (B) air-lift bioreactors displaying chromatograms used for UV-Vis monitoring of constituents at 280 nm (i) and 310 nm *(*ii). 4-HBAld, 4-hydroxybenzaldehyde; 4-HBAlc, 4-hydroxybenzyl alcohol; PCA, protocatechuic acid; pCoumA, *p*-coumaric acid; FA, ferulic acid.

Various sesquiterpenoids were identified in the ECM obtained from both the bioreactors. High-performance thin-layer chromatography-identified constituents were α-santalol, β-santalol, β-santalene, α-*trans*-bergamotol, epi-β-santalol and nuciferol (Table 3), while 7–10 constituents remained unidentifiable in the ECM (Fig. 3A and B).

**Table 3.** Identification and quantification of sesquiterpenoids from the air-lift bioreactors as inferred from HPTLC analyses.

Serial no.	Sesquiterpenoid constituents identified	Quantity (µg L^−1^ ECM)	
		2-L B. Braun reactor	10-L Nalgene vessel
1	α-Santalol	123 ± 17	131 ± 12
2	β-Santalol	345 ± 23	386 ± 27
3	β-Santalene	46 ± 6	53 ± 4
4	α-*trans*-Bergamotol	59 ± 7	73 ± 8
5	Epi-β-santalol	157 ± 28	186 ± 33
6	Nuciferol	91 ± 8	97 ± 11
7	Unidentified sesquiterpenoids	7	10

### Total yield of biomolecules (santalols, phenolics and AGP) from the ECM

**Figure 4.**
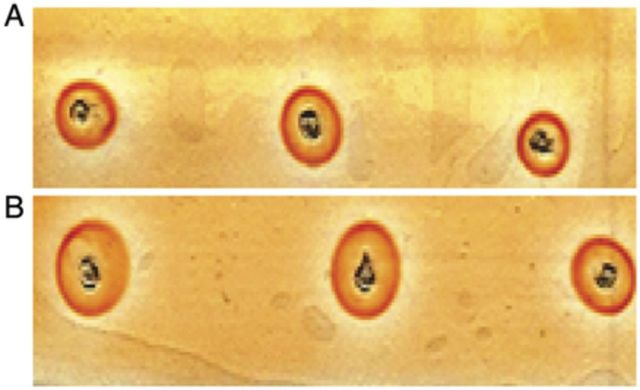
Quantification of AGPs in the ECM of 2-L (A) and 10-L (B) air-lift bioreactors performed by single radial gel diffusion assay carried out on histological slides.

High-performance thin-layer chromatography-based quantification indicated that the santalol yield from the ECM was comparable for both the air-lift reactors. In addition, results obtained from single radial agar diffusion assays (Fig. 4A and B) of the ECM demonstrated AGP yields of 33–39 mg L^−1^ for both the air-lift bioreactors. Furthermore, total yields of phenolics from ECM were quantified at 29–31 mg gallic acid equivalence L^−1^.

### Morphological evaluation of SEs

**Figure 5.**
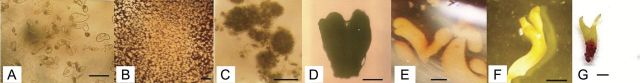
Microscopic observations of the single-cell suspension cultures used in the shake-flask cultures (scale bar: 100 µm) (A), the PEM used as inoculum (scale bar: 1 cm) (B), globular-shaped SEs (scale bar: 0.25 mm) (C), a single heart-shaped SE (scale bar: 0.5 mm) (D), a torpedo-shaped SE obtained post-cultivation (scale bar: 1 mm) (E), a matured cotyledonary stage SE (scale bar: 1 mm) (F) as obtained from the 10-L bioreactor. Weisner-stained SE showing development of lignin at the root pole (scale bar: 0.5 mm) of the bioreactor-grown SE (G).

Single-cell suspension cultures (Fig. 5A) from shake-flasks were the starting material for induction of embryogenesis to obtain the embryogenic inoculum. Matured SEs in various stages of development were obtained after 28 days culture of suspended PEMs in cultivation medium (Fig. 5B), although the majority were in globular stages. Different stages of SE development were observed in the final harvest, i.e. globular (Fig. 5C), heart-shaped (Fig. 5D) and torpedo stages (Fig. 5E), while the heart stage embryos were less frequent than others. The mature dicotyledonary embryos were ∼2–5 mm long (Fig. 5F). Thus normal SEs representative of a progressive somatic embryogenesis process were obtained at a high frequency. It was observed that the Wiesner (phloroglucinol-HCl) staining was located towards the root poles of the torpedo-stage matured SEs (Fig. 5G).

## Discussion

Air-lift bioreactors have wide applications due to their unique hydrodynamic characteristics, better oxygen transfer, good growth, reasonable mixing, less contamination due to the absence of moving parts and low operating cost due to simple design. Moreover, large-scale SE culture is an attractive alternative to the conventional method of plantation or cell culture. The use of large-scale liquid cultures and automation offers the potential to resolve manual handling of the various stages of micropropagation, thereby decreasing the production cost significantly. In addition, *in vitro*-grown plants are free from the effects of seasonal variations, microbial infestations and soil-borne contaminants that can affect the medicinal value of the harvested tissues. The successful production of SEs in bioreactors has been reported in a number of species (Paek *et al.* 2005). The growth and accumulation of secondary metabolites in cell cultures in bioreactors are influenced by various physical and chemical factors, like oxygen supply. Given that a metabolite is growth associated, a single-step reactor is sufficient to grow the cells and recover the molecules at the same time.

This investigation focused on the large-scale biomass production of sandalwood SEs with concomitant production of biomolecules of industrial importance. Therefore, we used a 10-L culture vessel as an air-lift bioreactor and compared the biomass and metabolite yield with that of a 2-L bioreactor. The simplicity of the 10-L air-lift bioreactor used in this study results in the low cost and efficient production of viable and matured SEs ranging from heart to torpedo stages. Furthermore, efficient top-to-bottom mixing makes this 10-L culture vessel and mixer system ideal for suspension cell culture applications. In fact, the agitation had a less pronounced effect on the 10-L vessel than the 2-L vessel, attributed to a larger culture volume, and hence the larger vessel was less prone to shearing stress (data not shown). The lowered pH of the ECM at harvest suggested the possible release of phenolics and sugar acids into the ECM during the growth phase, thereby influencing the pH. This investigation is a supplemental study on the production of peroxidase and AGP as by-products from SE cultivation in a 2-L air-lift bioreactor (Pal *et al.* 2003). Hence, we have not only obtained similar results to those reported before, but have also shown for the first time the production of santalol *in vitro* by means of HPTLC analysis. Furthermore, we demonstrated the production of phenolics in the ECM and confirmed their identities by RP-HPLC analysis.

In fact, air-lifts are generally considered unsuitable for high-density (>20 g L^−1^ DW) plant cell cultures due to the serious problems encountered during mixing and oxygen transfer (Dornenburg and Knorr 1995). However, we report SE biomass densities of 8.2–14 g L^−1^ by DW in this investigation, for both the bioreactors. Additionally, we show the accumulation of sesquiterpenoid alcohols in the ECM of SE cultures for the first time. In fact, the scale-up of plant cell suspensions to large culture volumes while maintaining their biosynthetic potential has been recognized as critical (Godoy-Hernandez *et al.* 2000).

In this investigation, the cell growth and biomass accumulation increased gradually with time and maximum biomass levels were reached after 28 days. Apart from the biomass, we screened the ECM for several metabolites such as phenolics, santalols and AGPs. In fact, phenolic acids are implicated in embryo development and are involved in alterations of the cell wall composition during differentiation and morphogenesis (Cvikrova *et al.* 1998). Additionally, hydroxycinnamates are involved in alterations of the cell wall composition (Lozovaya *et al.* 1996). Interestingly, we report the presence of hydroxycinnamates such as ferulic, protocatechuic and *p*-coumaric acids in the ECM of both the bioreactors, thus implying their role in SE development and maturation. Moreover, the presence of vanillin in the ECM of a 10-L-capacity bioreactor is notable. The Wiesner stain is known to react with cinnamaldehyde residues in lignin and the colour intensity roughly reflected the total lignin content. The observations in this study indicated lignin development towards the roots of SEs, thereby suggesting normal development of SEs. The staining reaction also indicated the biosynthesis of cinnamaldehydes in the SEs.

We further demonstrated the accumulation of AGPs in the ECM. Arabinogalactan proteins are cell wall proteoglycans that bind specifically to a synthetic probe, Yariv phenylglycoside, and are located at the plasma membrane, cell wall and in the media of cell cultures. Arabinogalactan proteins are present in both the periplasm and growth medium. Moreover, AGPs are known to promote somatic embryogenesis in cotton cell cultures (Poon *et al.* 2012). In fact, conditioned ECM is known to stimulate cell growth and accumulation of metabolites, such as anthocyanin production by strawberry suspension cells (Sakurai and Mori 1996). Probably, in the case of sandalwood SE suspension cultures in air-lift bioreactors, the inoculum carrying some amount of conditioned medium may be responsible for inducing the secretion of AGPs, santalols and phenolics.

## Conclusions

The most relevant and critical finding of this study is the development of a large-scale cultivation protocol optimized for a 10-L-volume bioreactor system. Secondary metabolites are currently being obtained commercially by extraction from whole plants or tissues. Moreover, cultured multipotent cambial meristematic cells have been identified as potential resources for the large-scale production of certain natural products (Roberts and Kolewe 2010). Large-scale SE culture in a bioreactor may provide an attractive alternative to the traditional method of sandalwood plantation. Although our results do not show significant increases in terms of metabolite production, the greater yield of biomass in 10-L bioreactors does indicate the commercial feasibility of the process for production of sandalwood biomolecules. Moreover, as a general rule, and due to their genetic stability, SEs are less prone to erratic metabolite production and display a lower sensitivity to shear stress than undifferentiated cells. Our results indicate that an efficient protocol for the mass production of sandalwood biomass can be achieved by a bioreactor-based cultivation of SEs and can simultaneously be used as a source of raw medicinal by-products of industrial importance.

## Sources of Funding

Funding for the presented investigation was obtained from the Ministry of Human Resources Development (MHRD), Government of India, India.

## Contributions by the Authors

B.B.M. carried out the experimental work described, while both B.B.M. and S.D. wrote the manuscript. S.D. obtained funding and managed the project.

## Conflicts of Interest Statement

None declared.

## Supporting Information

The following Supporting Information is available in the online version of this article –

**File 1.** Movie. Demonstrates the 2-L air-lift bioreactor (B. Braun) under running conditions supporting the growth of sandalwood somatic embryos.

**File 2.** Movie. Demonstrates the 10-L air-lift bioreactor (Nalgene) under running conditions supporting the growth of sandalwood somatic embryos.

Additional Information

## References

[PLT025C1] Bapat VA, Fulzele DP, Heble MR, Rao PS (1990). Production of sandalwood somatic embryo in bioreactors. Current Science.

[PLT025C2] Benencia F, Courreges MC (1999). Antiviral activity of sandalwood oil against Herpes Simplex Viruses-1 and -2. Phytomedicine.

[PLT025C3] Bommareddy A, Rule B, VanWert AL, Santha S, Dwivedi C (2012). α-Santalol, a derivative of sandalwood oil, induces apoptosis in human prostate cancer cells by causing caspase-3 activation. Phytomedicine.

[PLT025C4] Cvikrova M, Mala J, Eder J, Hrubcova M, Vagner M (1998). Abscisic acid, polyamines and phenolic acids in sessile oak somatic embryos in relation to their conversion potential. Plant Physiology and Biochemistry.

[PLT025C5] Das S, Das S, Mujib A, Pal S, Dey S (1998). El sandalo (*Santalum album*). Prensa Aromatica ANO.

[PLT025C6] Das S, Das S, Pal S, Mujib A, Sahoo SS, Ponde NR, Gupta S, Dey S (1999). A novel process for rapid mass propagation of the aromatic plant *Santalum album* in liquid media and bioreactor. Acta Horticulturae.

[PLT025C7] Dornenburg IH, Knorr D (1995). Strategies for the improvement of secondary metabolite production in plant cell cultures. Enzyme and Microbial Technology.

[PLT025C8] Eibl E, Werner S, Eibl D (2009). Disposable bioreactors for plant liquid cultures at litre-scale. Engineering in Life Sciences.

[PLT025C9] Georgiev MI, Weber J, Maciuk A (2009). Bioprocessing of plant cell cultures for mass production of targeted compounds. Applied Microbiology and Biotechnology.

[PLT025C10] Godoy-Hernandez GC, Vazquez-Flota FA, Loyola-Vargas VM (2000). The exposure to trans-cinnamic acid of osmotically stressed *Catharanthus roseus* cells cultures in 14-l bioreactor increases alkaloid accumulation. Biotechnology Letters.

[PLT025C11] Harborne AJ (1998). Phytochemical methods. *A guide to modern techniques of plant analysis*.

[PLT025C12] Howes MJR, Simmonds MSJ, Kite GC (2004). Evaluation of the quality of sandalwood essential oils by gas chromatography-mass spectrometry. Journal of Chromatography A.

[PLT025C13] Huang T-K, McDonald KA (2009). Bioreactor engineering for recombinant protein production in plant cell suspension cultures. Biochemical Engineering Journal.

[PLT025C14] IUCN (2012). www.iucnredlist.org.

[PLT025C15] Julkunen-Tiitto R, Häggman H, Aphalo P, Lavola A, Tegelberg R, Veteli T (2005). Constraints of UV-B radiation in deciduous trees. Environmental Pollution.

[PLT025C16] Kitazawa K, Tryfona T, Yoshimi Y, Hayashi Y, Kawauchi S, Antonov L, Tanaka H, Takahashi T, Kaneko S, Dupree P, Tsumuraya Y, Toshihisa Kotake T (2013). β-Galactosyl Yariv reagent binds to the β-1,3-galactan of arabinogalactan proteins. Plant Physiology.

[PLT025C17] Kolewe ME, Gaurav V, Roberts SC (2008). Pharmaceutically active natural product synthesis and supply via plant cell culture technology. Molecular Pharmaceutics.

[PLT025C19] Lloyd DG, McCown BH (1981). Commercially-feasible micropropagation of Mountain laurel, *Kalmia latifolia*, by use of shoot tip culture. The International Plant Propagators Society.

[PLT025C20] Lozovaya V, Gorshkova T, Yablokova E, Zabotina O, Ageeva M, Rumyantseva N, Kolesnichenko E, Waranyuwat A, Widholm J (1996). Callus cell wall phenolics and plant regeneration ability. Journal of Plant Physiology.

[PLT025C21] Matkowski A (2008). Plant *in vitro* culture for the production of antioxidants—a review. Biotechnology Advances.

[PLT025C23] Misawa M, Komamine A, Misawa M, DiCosmo F (1991). Research activities in Japan. Plant cell culture.

[PLT025C24] Misra BB, Dey S (2012). Comparative phytochemical analysis and antibacterial efficacy of *in vitro* and *in vivo* extracts from East Indian sandalwood tree (*Santalum album* L.). Letters in Applied Microbiology.

[PLT025C25] Ochi T, Shibata H, Higuti T, Kodama K, Kusumi T, Takaishi Y (2005). Anti-*Helicobacter pylori* compounds from *Santalum album*. Journal of Natural Products.

[PLT025C26] Paek KY, Chakrabarty D, Hahn EJ, Kathrine AEH, Walter P (2005). Application of bioreactor system for large scale production of horticultural and medicinal plants. Liquid culture systems for in vitro plant propagation.

[PLT025C27] Pal S, Das S, Dey S (2003). Peroxidase and arabinogalactan protein as by-products during somatic embryo cultivation in air-lift bioreactor. Process Biochemistry.

[PLT025C28] Paul M, Ma JK-C (2011). Plant-made pharmaceuticals: leading products and production platforms. Biotechnology and Applied Biochemistry.

[PLT025C29] Pomar F, Merino F, Ros Barceló A (2002). O-4-Linked coniferyl and sinapyl aldehydes in lignifying cell walls are the main targets of the Wiesner (phloroglucinol-HCl) reaction. Protoplasma.

[PLT025C30] Poon S, Heath RL, Clarke AE (2012). A chimeric arabinogalactan protein promotes somatic embryogenesis in cotton cell culture. Plant Physiology.

[PLT025C31] Rangaswamy NS, Rao PS (1963). Experimental studies on *Santalum album* L.—establishment of tissue culture of endosperm. Phytomorphology.

[PLT025C32] Roberts S, Kolewe ME (2010). Plant natural products from cultured multipotent cells. Nature Biotechnology.

[PLT025C33] Sachan A, Ghosh S, Mitra A (2004). An efficient isocratic separation of hydroxycinnamates and their corresponding benzoates from microbial and plant sources by HPLC. Biotechnology and Applied Biochemistry.

[PLT025C34] Saklani A, Kutty SK (2008). Plant-derived compounds in clinical trials. Drug Discovery Today.

[PLT025C35] Sakurai M, Mori T (1996). Stimulation of anthocyanin synthesis by conditioned medium produced by strawberry suspension cultures. Journal of Plant Physiology.

[PLT025C36] Tabata M, Fujita Y, Zaitlin M, Day P, Hollaender A (1985). Production of shikonin by plant cell cultures. Biotechnology in plant science. Relevance to agriculture in the eighties.

[PLT025C37] Weathers PJ, Towler MJ, Xu J (2010). Bench to batch: advances in plant cell culture for producing useful products. Applied Microbiology and Biotechnology.

[PLT025C38] Wilson SA, Roberts SC (2012). Recent advances towards development and commercialization of plant cell culture processes for the synthesis of biomolecules. Plant Biotechnology Journal.

[PLT025C39] Xu J, Ge X, Dolan MC (2011). Towards high-yield production of pharmaceutical proteins with plant cell suspension cultures. Biotechnology Advances.

