# Supplementary material for: Culture of East Indian sandalwood tree somatic embryos in air-lift bioreactors for production of santalols, phenolics and arabinogalactan proteins
Source: AoB Plants. 2013 May 6;5:plt025. doi: 10.1093/aobpla/plt025 (PMC4455360; doi:10.1093/aobpla/plt025)
Supplement: Additional Information [file supp_5_plt025_index.html]

Culture of East Indian sandalwood tree somatic embryos in air-lift bioreactors for production of santalols, phenolics and arabinogalactan proteins — Additional Information 

# Culture of East Indian sandalwood tree somatic embryos in air-lift bioreactors for production of santalols, phenolics and arabinogalactan proteins

## Additional Information

**Files in this Data Supplement:**

- Additional Information - Additional Information Movie 1
- Additional Information - Additional Information Movie 2
